# Supplementary material for: Profile of Children with Undernutrition Admitted in Two Secondary-Level Hospitals in Maputo City, Mozambique
Source: Nutrients. 2024 Apr 4;16(7):1056. doi: 10.3390/nu16071056 (PMC11013278; doi:10.3390/nu16071056)
Supplement: Supplementary file 1 [file nutrients-16-01056-s001.zip › Table S1. Nutritional assessment classification in children and adults.pdf]

Table S1. Nutritional assessment classification in children and adults [24,25].

Outcomes	Anthropometric measures and cut points	Nutritional status
Children		
BMI-for-age	BAZ < -2 SD	Thinness
	BAZ < -3 SD	Severely thinness
Wasting	WHZ < -2 SD	Wasted
	WHZ < -3 SD	Severely wasted
Stunting	HAZ < -2 SD	Stunted
	HAZ < -3 SD	Severely stunted
Adults (caretakers)		
BMI	BMI < 18.5	Underweight
	18.5 ≥ BMI >24.9	Normal
	25 ≥ BMI >29.9	Overweight
	BMI ≥ 30	Obese
