# Supplementary material for: Profile of Children with Undernutrition Admitted in Two Secondary-Level Hospitals in Maputo City, Mozambique
Source: Nutrients. 2024 Apr 4;16(7):1056. doi: 10.3390/nu16071056 (PMC11013278; doi:10.3390/nu16071056)
Supplement: Supplementary file 1 [file nutrients-16-01056-s001.zip › Table S2. The wealth index asset items included for the socioeconomic status determination.pdf]

Table S2. The wealth index asset items included for the socioeconomic status determination [6].

Category	Variable	Wealthier	Poorer
**Mobility**	Mobility (if has any of vehicle for example: bicycle, motorized, car)	1-yes	0- no
**Household characteristics**	Electricity	1-yes	0- no
	Freezer	1-yes	0- no
	Cell phone	1-yes	0- no
	Television	1-yes	0- no
**Money saving**	Bank account (also includes mobile money accounting for example mpesa, mkesh)	1-yes	0 – no
**Household utilities and other**	Type of water supply	1-pipped	0-fountain or unprotected well
	Type of toilet	1-sink with and without flush	0-none, latrine, well
	Type of flooring	1-cement, bricks	0-ground
	Type of walls/house	1-concrete blocks	0-zinc sheets, reed, other rudimentar
	Type of roof	1-cement	0-zinc sheets
	Type of source for cooking	1-electricity, gas and a combination with other sources (where electricity or gas is included)	0-wood, coal
	Sharing toilet (with other families)	1-no	0-yes
	Location of the toilet	1-inside	0-outside of the house
	Land ownership and/or small piece of land for agriculture (includes in their home)	1-yes	0- no
